# One-Pot and Green Preparation of *Phyllanthus emblica* Extract/Silver Nanoparticles/Polyvinylpyrrolidone Spray-On Dressing

**DOI:** 10.3390/polym14112205

**Published:** 2022-05-29

**Authors:** Whijitra Suvandee, Veerawat Teeranachaideekul, Nutjaree Jeenduang, Patcharakamon Nooeaid, Arthit Makarasen, Laemthong Chuenchom, Supanna Techasakul, Decha Dechtrirat

**Affiliations:** 1 Department of Materials Science, Faculty of Science, Kasetsart University, Bangkok 10900, Thailand; whijitra.s@ku.th; 2 Department of Pharmacy, Faculty of Pharmacy, Mahidol University, Bangkok 10400, Thailand; veerawat.tee@mahidol.ac.th; 3 School of Allied Health Sciences, Walailak University, Nakhon Si Thammarat 80160, Thailand; nutjaree.je@wu.ac.th; 4 Division of Polymer Materials Technology, Faculty of Agricultural Product Innovation and Technology, Srinakharinwirot University, Nakhon-Nayok 26120, Thailand; patcharakamon@g.swu.ac.th; 5 Laboratory of Organic Synthesis, Chulabhorn Research Institute, Bangkok 10210, Thailand; arthit@cri.or.th (A.M.); supanna@cri.or.th (S.T.); 6 Division of Physical Science and Center of Excellence for Innovation in Chemistry, Faculty of Science, Prince of Songkla University, Songkhla 90110, Thailand; laemthong.c@psu.ac.th; 7 Specialized Center of Rubber and Polymer Materials for Agriculture and Industry (RPM), Faculty of Science, Kasetsart University, Bangkok 10900, Thailand

**Keywords:** green synthesis, spray-on dressing, silver nanoparticle, wound healing, *Phyllanthus emblica*

## Abstract

A spray-on wound dressing has many benefits, including easy and quick administration to broad and uneven wounds, better interface with the wound site, adhesion without additional dressing, and multiple applications in a portable package. By limiting direct contact with the wound site, such a design can prevent wound damage during treatment. This study revealed a simple, one-pot synthesis of spray-on wound dressing relying on polyvinylpyrrolidone solution incorporating silver nanoparticles as a broad-spectrum antibacterial agent and wound-healing antioxidant *Phyllanthus emblica* extract. Silver nanoparticles were synthesized in situ using *Phyllanthus emblica* extract as a biogenic reducing agent. Polyvinylpyrrolidone was employed as a film-forming agent to create an adhesive hydrogel-based dressing matrix to provide moisture and establish a shielding barrier for the wound bed as well as to regulate the release of fruit extract. In vitro tests revealed that the produced dressing film had a controlled release of the fruit extract, high antioxidant activity, and a good antibacterial action against *S. aureus*, *P. aeruginosa*, *E. coli*, and MRSA. Additionally, a biocompatibility study has shown that both human fibroblasts and keratinocytes are unaffected by the dressing film. Based on established findings, the current spray-on solution might be a potential option for antibacterial wound dressing.

## 1. Introduction

Conventional wound dressings, such as cotton wool, bandages, lint, gauze, and plasters, are often used as main or supplementary dressings for clean and dry wounds with low discharge levels to safeguard the site from contamination [1]. Conventional dressings, on the other hand, do not offer a moist environment for the wound. They are normally prefabricated and need a layer of adhesive covering for attachment. They are also difficult to put on wounds with uneven forms and wide regions. Such dressings typically need replacement often to avoid fluid buildup, which is unpleasant, disrupts the healing skin, and may boost the risk of contamination [2]. Modern dressings, on the other hand, are meant to keep the wound moisturized and encourage healing as well as protect it from infection. Semi-porous films, semi-porous foams, hydrogels, hydrocolloids, and alginates are some examples of modern wound dressings. Nonetheless, concerns about the aforementioned dressings exist, such as user-friendliness, flexibility, and transportability [3]. Such problems can be handled by inventing a quick gel-forming spray that can be put directly on the wound without coming into contact with hands or cotton swabs, lowering the risk of infection or contaminants. Typically, these sprays generate thin, adhesive coatings, eliminating the risk of further trauma while dressing or re-dressing the wound [4]. Intimate engagement with the injured area, simple and fast treatment to vast and uneven wounds, minimal discomforts during treatment, many uses with a portable container, and ultimately improved patient compliance are all advantages of this approach [5]. In addition to promoting tissue repair, the spray-on wound dressing can deliver drugs that limit the chance of infection and inflammation. Drug dosages in a film-forming spray can also be changed depending on the volume of solution in each spray [6]. In comparison to patches, a thin film created from the spray is effortless to wash off using just water [7] and can improve patient comfort while moving [8,9]. Due to their ease of use and benefits, spray-on dressings are becoming progressively popular [2,3,4,5,6,7,10,11,12,13].

Antimicrobial drugs serve a critical function in bacterial burden reduction. Silver nanoparticles (AgNPs) have been shown to successfully limit the growth of both common and drug-resistant bacterial strains in many studies. It exhibits broad antibacterial efficacy against both Gram-positive and Gram-negative bacteria, with less bacterial resistance formation [14]. Lately, nanotechnology has made it possible to produce many kinds of AgNPs. Because of their small size, AgNPs have a higher surface area-to-mass ratio, which allows them to make better contact with bacteria and have a stronger antibacterial effect as a result [15]. It is worth noting that the typical chemical reducing agents used in AgNP synthesis are toxic and could cause health problems. As a consequence, researchers have documented the utilization of plant extracts in the green synthesis approach to manufacture and stabilize metallic nanoparticles [16]. Many publications have detailed the addition of AgNPs into dressings in varying forms, such as foams, hydrogels, and polymeric films, each claiming to have different advantages but all claiming silver’s bactericidal effectiveness [17,18,19,20,21]. In addition to assisting in the creation of metallic nanoparticles, plant extracts are a good source of bioactive chemicals, with a wide diversity of secondary metabolites that have a wide range of pharmacological effects.

*Phyllanthus emblica* is a tropical and subtropical plant used in folk medicine for a long time to treat common colds and fevers, sore throats, coughs, dry mouth, diarrhea, inflammation, and wounds, either alone or in combination with other components [22,23]. The effect of *P. emblica* extract on wound healing has been documented, including mechanisms involving collagen growth, extracellular matrix (ECM) protein synthesis, and antioxidant status [24,25,26]. *P. emblica* was discovered to have strong antioxidant and anti-inflammatory properties in previous investigations. Polyphenols, flavonoids, tannins, and vitamins are the main active phytochemicals typically found in *P. emblica*, particularly the fruits. Polyphenols, including gallic acid, ellagic acid, and chebulagic acid, are thought to be the main active ingredients behind *P. emblica*’s anti-inflammatory and antioxidant properties [27,28]. Polyphenols have been shown to be potent antioxidizing agents [29,30] due to their abilities to donate/accept electrons and delocalize the unpaired electron inside their aromatic structure. For wound healing, antioxidants are thought to help with tissue repair by assisting in the defense against oxidative stress and inflammation [31]. To have a long-lasting effect of antioxidants, the incorporation of antioxidants into sustained-release polymeric systems is one of the recognized approaches for extending antioxidant effects.

Polyvinylpyrrolidone (PVP) is a water-soluble and biodegradable polymer. PVP has various distinct physical and chemical characteristics, including being chemically inert, colorless, temperature-resistant, and pH-stable [32]. It also has excellent solubility in solvents of various polarities and superior adhesive qualities. As it is non-toxic and biocompatible, PVP is used extensively in the pharmaceutical and biomedical industries [33]. It has been employed in the development of a variety of drug delivery systems, including oral, ophthalmic, topical, and transdermal delivery in a variety of formats, such as particles, hydrogels, and fibers [32,34]. PVP can also be used to create clear, thin, and well-distributed films [6]. Since PVP keeps skin moist and prevents scab development, it can be applied as the core element in topical coverings for skin regeneration and wound dressings [35,36]. PVP is also acknowledged as one of the most effective polymeric stabilizers for silver nanoparticles [37]. 

**Scheme 1:**
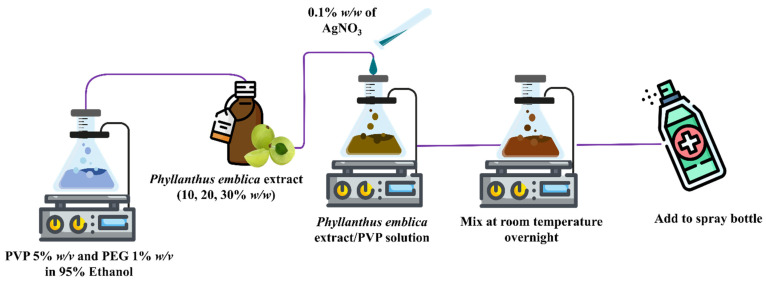
Schematic illustration of a one-pot preparation protocol for *P. emblica* extract/silver nanoparticles/polyvinylpyrrolidone spray solution.

Accordingly, this study set out to create a simple methodology for manufacturing a spray-on wound dressing with silver nanoparticles as a broad-spectrum antibacterial agent and *Phyllanthus emblica* extract as antioxidants to promote wound healing. The spray-on solution was made in one pot using a natural fruit extract made from *Phyllanthus emblica* for the green synthesis of silver nanoparticles (Scheme 1). The adhesive hydrogel film will form quickly after being sprayed and act as a protective layer to keep moisture in the wound bed and keep germs out. Furthermore, the produced gel film serves as a polymeric-based matrix in this scenario, controlling the discharge of the fruit extract and extending its antioxidant effects. A variety of procedures were used to characterize the prepared dressing. To illustrate the possibility of using the synthesized materials as wound dressings, in vitro release, antioxidant, antibacterial, and cytotoxicity tests were conducted. 

## 2. Materials and Methods

### 2.1. Materials

*Phyllanthus emblica* fruit extract was obtained from Chemipan Corporation Co., Ltd. (Bangkok, Thailand). Polyvinylpyrrolidone K30 (MW~40,000) and PEG (Mw~400) were attained from TCI chemicals (Tokyo, Japan). Silver nitrate was from Alfa Aesar (Kandel, Germany). Folin–Ciocalteu reagent and 2,2-diphenyl-1-picrylhydrazyl (DPPH) were purchased from Sigma-Aldrich (Burlington, MA, USA). (3-(4,5-dimethylthiazol-2-yl)-5-(3-carboxymethoxyphenyl)-2-(4-sulfophenyl)-2H-tetrazolium inner salt was obtained from Promega Corporation (Madison, WI, USA). Dulbecco’s modified Eagle’s medium (DMEM, Gibco^®^) with 10% fetal bovine serum (FBS, Gibco^®^), 1 mM sodium pyruvate (Gibco^®^), and penicillin/streptomycin (Gibco^®^) were supplied by Life Technologies (Carlsbad, CA, USA). Buffer salts and organic solvents were of analytical grade from Merck (Darmstadt, Germany).

### 2.2. Preparation of P. emblica Extract/Silver Nanoparticles/Polyvinylpyrrolidone Solution

The polymer solution was prepared by dissolving polyvinylpyrrolidone (5% *w*/*v*) and polyethylene glycol (1% *w*/*v*) in 95% ethanol. *Phyllanthus emblica* extract was added into the polymeric solution at 10%, 20%, and 30% (*w*/*w* to the polymer content). After that, AgNO_3_ (0.1% *w*/*w* to the polymer content) was gradually added to the *Phyllanthus emblica* extract/polymer solution and mixed thoroughly at room temperature overnight. 

### 2.3. Characterization of the Spray-On Solution and Film

#### 2.3.1. Evaporation Time

The evaporation time of each spray formulation was evaluated in terms of weight loss percentage over time. The experiment was performed at an ambient temperature of 32 ± 1 °C and relative humidity of 60% by spraying the solution onto a glass Petri dish. The real-time change in weight was then monitored using a computer-controlled electronic analytical balance (ME204, Mettler Toledo, Columbus, OH, USA) with an accuracy of 0.0001 g.

#### 2.3.2. UV/Vis Spectra Analysis

The absorption spectrum of the spray solution was obtained using a UV/Vis spectrophotometer (UV-2600i, Shimadzu Corp., Kyoto, Japan). Spectra were recorded between 320 and 700 nm.

#### 2.3.3. XRD Analysis

X-ray diffraction was used to identify the crystalline phase of silver nanoparticles in the sample. Measurement was conducted on a Bruker D8 ADVANCE X-ray diffractometer (Bruker, Karlsruhe, Germany) using Ni filtered CuK_𝛼_ radiation (λ = 1.5406 Å) generated at a current of 30 mA and a voltage of 30 kV. The XRD patterns were recorded from 15° to 80° at a scanning speed of 5°/min.

#### 2.3.4. Particle Size and Zeta Potential Analysis

The particle size of silver nanoparticles was evaluated by a field-emission transmission electron microscope (FE-TEM, JEM-3100F, JEOL, Tokyo, Japan) operated at a voltage of 300 kV. The particle size distribution and average diameters of silver nanoparticles were determined by analyzing 100 particles from the TEM images using ImageJ software (NIH). Zeta potential of the colloidal particles was evaluated by zeta potential analyzer (Zetasizer Ultra, Malvern Instruments Ltd., Worcestershire, UK).

#### 2.3.5. FTIR Analysis

FTIR analysis was carried out to identify the chemical characteristics of the spray-on films. The spectra were recorded using a Fourier transform infrared spectrometer (Spectrum One FTIR, Perkin Elmer, Waltham, MA, USA) from 4000 to 500 cm^−1^ at a resolution of 4 cm^−1^ and a total of 64 scans.

#### 2.3.6. Film Morphology Analysis

The sample was cast onto a clean glass slide and placed on a sample stub with double-sided mounting tape. The morphology of the film was visualized using a scanning electron microscope (Quanta 450, FEI, Eindhoven, the Netherlands) operated at an accelerating voltage of 15 kV.

### 2.4. In Vitro Release

In vitro release of *Phyllanthus emblica* extract from the dressing film was carried out using 12 mL Franz diffusion cells with a diffusion area of 2.54 cm^2^. To simulate skin surface temperature, the experiment was conducted at 32 ± 0.5 °C using PBS (pH 7.4) as a receptor medium. The precast spray-on dressing film was mounted onto a cellulose acetate membrane and then inserted between the donor and receptor chambers. Throughout the experiment, the receptor medium was stirred continuously. At predetermined time intervals of 5, 10, 15, 20, 25, 30, 60, 90, 120, 240, 360, and 480 min, 1 mL of the medium was discharged and directly refilled with an equivalent amount of a fresh medium. The collected samples were thereafter analyzed by a UV/vis spectrophotometer. 

To explain the release mechanism, the initial 60% of the cumulative release was fitted to the following the Ritger–Peppas kinetic equation:

M_t_/M_∞_ = kt^n^

In this equation, M_t_/M_∞_ is the cumulative release of the extract at time t, k is the kinetic constant, and n is the diffusion exponent, which classifies the release mechanism. When n is equal to or less than 0.5, the release mechanism is considered a Fickian diffusion (diffusion through a non-swollen matrix). When n falls between 0.5 and 1, the release mechanism follows an anomalous non-Fickian transport (release through both diffusion and dissolution/swelling of the matrix). When n is equal to 1.0, the release mechanism is the case II transport (release through dissolution/swelling of the matrix) [38].

### 2.5. Determination of Total Phenolic Content

The total phenolic content of the spray-on dressing solutions was evaluated using the Folin–Ciocalteu method with slight modifications. The total phenolic content was expressed as mg of gallic acid equivalence per gram of sample (mg GAE/g sample). The test was carried out in triplicate and reported as the mean ± standard deviation.

### 2.6. In Vitro Antioxidant Test

The DPPH radical scavenging activity of the spray-on dressing solutions was determined using the protocol previously described by Das and Goyal with slight modifications [39]. Briefly, 100 µL of 0.4 mM methanolic DPPH solution and 100 µL of test samples were thoroughly mixed and kept at 37 °C for 30 min in the dark. The scavenging activity was evaluated by measuring the absorbance at 517 nm using a microplate reader, after which the percentage of DPPH radical scavenging activity was calculated using the following equation:

$$\mathrm{DPPH}\;\mathrm{radical}\;\mathrm{scavenging}\;\mathrm{activity}\;\left(\%\right)=\frac{\left[A\;\mathrm{control}-A\;\mathrm{sample}\right]}{\left[A\;\mathrm{control}\right]}\times 100$$

In this equation, A _sample_ and A _control_ refer to the absorbance of DPPH solution with and without sample, respectively.

### 2.7. In Vitro Antibacterial Test

The antibacterial activity was determined by the disk diffusion test. Four representative bacterial strains typically found in wound infections, including *Staphylococcus aureus* (*S. aureus*, TISTR 517), methicillin-resistant *Staphylococcus aureus* (MRSA, TISTR 142), *Pseudomonas aeruginosa* (*P. aeruginosa*, TISTR 1467), and *Escherichia coli* (*E. coli*, TISTR 887), were incubated on Mueller–Hinton agar (MHA) at 37 ℃ for 24 h. The bacterial suspension with a turbidity equivalent to the 0.5 McFarland standard was prepared and spread over the Mueller–Hinton agar plate. The test samples were prepared by impregnation of the spray solutions onto 6 mm disks of Whatman filter paper, which were then mounted on the agar plates and incubated at 37 ℃ for 24 h. Afterward, antibacterial efficacy was determined by measuring the zone of inhibition (mm). The tests were carried out in triplicate and reported as mean diameter ± standard deviation.

### 2.8. In Vitro Cytotoxicity Test

In vitro cytotoxicity on human dermal fibroblast (HDFa) (ATCC^®^ PCS-201-012™) and immortalized human keratinocyte (HaCat) (ATCC^®^ Number PCS-200-011™, 300493, CLS, Germany) was carried out using the (3-(4, 5-dimethylthiazol-2-yl)-5-(3-carboxymethoxyphenyl)-2-(4-7sulfophenyl)-2H-tetrazolium) (MTS) assay. HDFa and HaCat cells were cultured in Dulbecco’s modified Eagle’s medium (DMEM, Gibco^®^, Life Technologies, USA) with 10% fetal bovine serum (FBS, Gibco^®^), 1 mM sodium pyruvate (Gibco^®^), 100 U/mL penicillin, and 100 µg/mL streptomycin at 37 °C and 5% CO_2_. One day before the experiment, the cultured cells were seeded into a 96-well plate (10,000 cells/well for fibroblast and 8000 cells/well for keratinocyte) and incubated for 24 h. After that, the medium was exchanged with the test substances at various doses (0.63, 1.25, and 2.5 mg/mL), and the cells were incubated for another 24 h. The test sample was then removed, and the MTS reagent was added and incubated at 37 °C for 3 h. The solution containing soluble formazan product was collected, and the optical density (OD) of the obtained solution was measured at 490 nm. The percentage of cell viability was then calculated using the following equation:

$$\%\;\mathrm{cell}\;\mathrm{viability}=\frac{\left[\mathrm{OD}\;\mathrm{sample}\right]}{\left[\mathrm{OD}\;\mathrm{negative}\;\mathrm{control}\right]}\times 100$$

### 2.9. Statistical Analysis

All experimental data are presented as mean ± standard deviation (SD). To compare the means by factor levels, one-way analysis of variance (one-way ANOVA) was employed, with *p*-values < 0.05 deemed significant.

## 3. Results and Discussion

### 3.1. Characterization of P. emblica Extract/Silver Nanoparticles/Polyvinylpyrrolidone Solution

#### 3.1.1. Evaporation Rate

**Figure 1:**
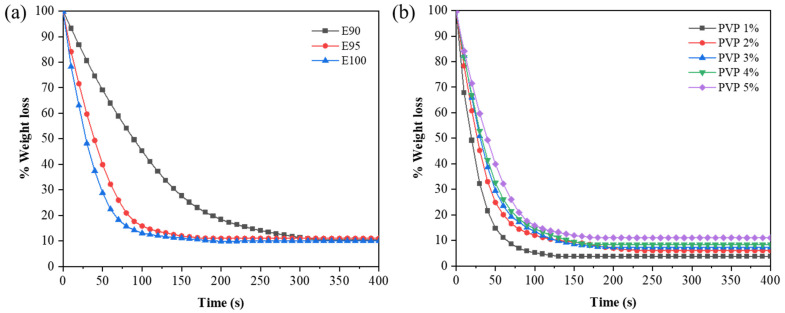
Evaporation rates of PVP solutions at different (**a**) solvent ratios and (**b**) polymer concentrations.

The evaporation rate is a crucial aspect to consider when formulating the spray-on dressing since it determines how quickly the film develops after being applied. The time it takes for the solvent to evaporate and the film to develop may be used to identify the evaporation rate. In this study, the evaporation rate was determined by tracking the weight loss % over time. This was accomplished by spraying the dressing solution onto a glass Petri dish and then using a computer-controlled electronic analytical balance to evaluate the real-time change in weight. In this study, the effects of the solvent ratio and polymer concentration on the evaporation rate were investigated. Since ethanol and water are both safe and often used in pharmaceutical preparations, they were chosen as carrier solvents for the spray formulations. The solvent solution that thoroughly dissolves the polymer while also allowing for rapid evaporation was optimized by adjusting the ethanol and water mixture ratio. Spray solutions of 5% (*w*/*v*) PVP in three distinct solvent systems (90%, 95%, and 100% ethanol) were examined to see whether the solvent ratio had an impact on the evaporation rate. The sprayed solution’s weight loss % was tracked and illustrated as a function of time. The steepness of the curve on the plot may be used to determine the evaporation rate. Figure 1a shows how weight loss begins quickly and then steadily reduces until it reaches a plateau. It indicates that the rate of solvent evaporation is initially fast owing to a large amount of volatile organic solvent lost but later slows down when the polymeric gel sets and reduces solvent evaporation. When compared to the other two spray solutions, the one produced with 100% ethanol showed the fastest deterioration. Despite having the quickest evaporation rate, the resulting film was relatively dry and brittle, likely due to the absence of water in the solvent solution. The evaporation rate decreased as the water concentration in the binary solvent solution increased because water is less volatile than ethanol. The spray evaporated almost as rapidly with 5% water in the solvent as it did with 100% ethanol, but the resulting gel coating was transparent, moist, and soft. As a result, the most suitable solvent system was 95% ethanol, which was also utilized to create the spray. 

Aside from the solvent ratio, the polymer concentration was changed to find the best quantity of polymer for quickly forming a gel film. In 95% ethanol, the polymer content ranged from 1% to 5% *w*/*v*. The resulting solutions were all transparent at all polymer concentrations, indicating that the polymer could be fully dissolved. When the polymer concentration is increased from 1% to 2%, the rate of evaporation reduces, as seen in Figure 1b. Thus, the concentration of polymer influences the rate of evaporation. When the polymer concentration is greater than 2%, however, the impact appears to be less apparent. The polymeric gel that forms after being sprayed is responsible for this outcome. The spray droplets are too tiny when the polymer concentration is too low. As a result, they may be unable to spread throughout the substrate and may dry quickly. With a greater polymer content, the resulting film becomes more uniform and serves as a superior moisture barrier within the gel. As a result, the rate of evaporation is lower [5]. The spray with 5% *w*/*v* PVP produced a more uniform film with an equivalent evaporation rate than the other sprays with lower PVP content; hence, it was selected and utilized to make the spray-on dressing with silver nanoparticles and *Phyllanthus emblica* extract. Since the skin is covered by a significant number of skin pores through which the solvent may be absorbed, the evaporation rate on the actual skin may be quicker than the test carried out on a simple glass plate. Furthermore, an increase in skin temperature enhances solvent evaporation, reducing film drying time.

#### 3.1.2. UV/Vis Spectra Analysis

**Figure 2:**
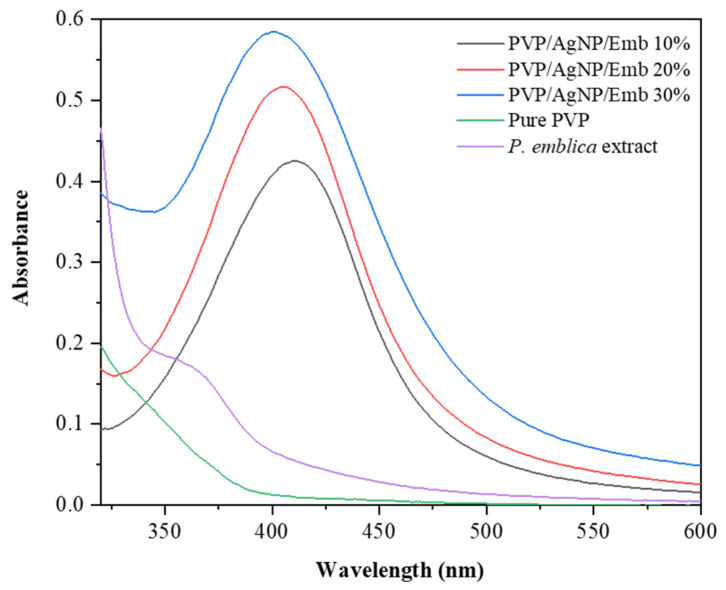
UV/VIS absorption spectra of PVP, *Phyllanthus emblica* extract, and dressing spray containing different amounts of *Phyllanthus emblica* extract.

In this study, silver nanoparticles were created utilizing a green chemical approach with *Phyllanthus emblica* extract. Kannaujia et al. previously proposed the mechanism for the synthesis [40]. One of the major chemical constituents in the *Phyllanthus emblica* fruit extract, ascorbic acid, acts as a green reducing agent, converting silver ions into metallic silver atoms, which then aggregate to create silver nanoparticles. Ascorbic acid will turn into dehydroascorbic acid after the reduction process. UV/VIS spectrophotometry can be used to validate the formation of silver nanoparticles. Owing to the intense surface plasmon resonance (SPR) of the colloidal silver nanoparticles, the dressing spray comprising varying concentrations of *Phyllanthus emblica* extract exhibits characteristic broad absorption bands ranging from 320 to 600 nm, as observed in the absorption spectra (Figure 2). The size of the produced particles is expected to be lower than 100 nm, as the absorption maxima do not exceed 500 nm. The peak intensity rises as the amount of extract loaded increases. With the addition of the biogenic reducing agent in the *Phyllanthus emblica* extract, the absorbance of the SPR peaks increased, implying an increase in the number of silver nanoparticles. Furthermore, an increase in the extract loading quantity causes a blue shift of the highest absorption peaks towards shorter wavelengths due to the reduction in particle size. This might be because of an increase in the amount of nanoparticle nucleation seeds, leading to the establishment of smaller particles [41].

#### 3.1.3. XRD Analysis

**Figure 3:**
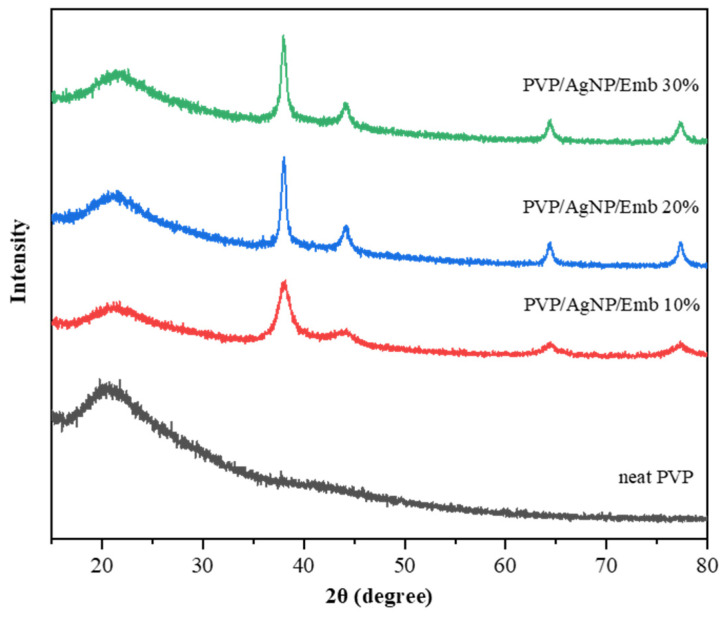
X-ray diffraction patterns of spray-on dressing films loaded with different amounts of *Phyllanthus emblica* extract and pure PVP film.

To corroborate the presence of silver nanoparticles, X-ray diffraction was employed. The diffraction pattern of the neat PVP presented typical broad peak of the amorphous structure at 2θ of 21.3°, while the spray-on dressing showed a broad peak of PVP at around 21.2°, and the other four individual diffraction peaks at 38.2°, 44.2°, 64.3°, and 77.2°, which are consistent with the (111), (200), (220), and (311) planes of the face-centered cubic crystal structure of silver, as illustrated in Figure 3. This demonstrated that the recommended procedure could successfully produce silver nanoparticles. As previously indicated in the case of the UV/VIS absorption spectra, the peak intensity of silver increases as the loading quantity of the extract increases owing to an increase in the number of silver nanoparticles. 

#### 3.1.4. Particle Size and Zeta Potential Analysis

**Figure 4:**
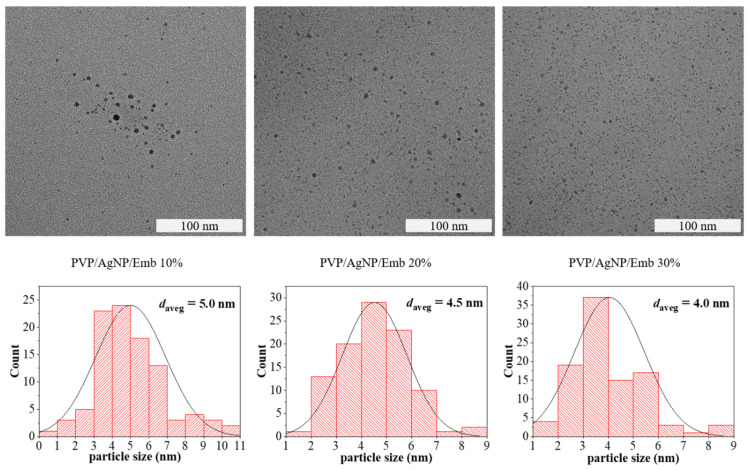
TEM images of silver nanoparticles presented in three different formulations of the dressing spray at 100,000× magnification and the particle size histograms.

The presence of silver nanoparticles and their sizes were confirmed using a transmission electron microscope. For all of the spray compositions, the produced nanoparticles were spherical, as shown in Figure 4. Analysis of 100 particles from TEM micrographs was used to determine the distribution and average sizes of silver nanoparticles, with the results displayed in histograms. For the spray-on dressing loading with 10%, 20%, and 30% *Phyllanthus emblica* extract, the average diameters of the existing particles were observed at 5.0 ± 0.6, 4.5 ± 0.6, and 4.0 ± 0.8 nm, respectively. Compared to silver nanoparticles of greater size, particles having a diameter of roughly 5 nm have previously been found to have the best and quickest bactericidal efficacy [42]. The zeta potential of colloidal AgNPs was evaluated, and the mean zeta potential values for spray-on dressing loaded with 10%, 20%, and 30% *Phyllanthus emblica* extract were found to be −29.2, −31.4, and −33.2 mV, respectively, indicating that the synthesized nanoparticles are stable. These findings are comparable to previously published values for PVP-capped AgNPs [43,44,45]. The creation of H-bonded compounds in the pyrrolidone ring with water molecules [46] as well as the capping impact of the components from *Phyllanthus emblica* fruit extract [47] may explain the negative charge.

#### 3.1.5. FTIR Analysis

**Figure 5:**
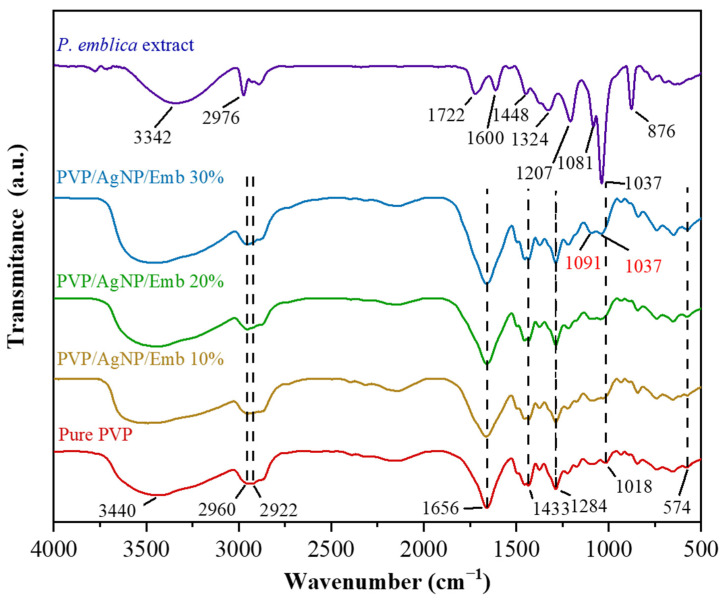
FTIR spectra of *Phyllanthus emblica* extract, composite spray-on dressing films loaded with different amounts of *Phyllanthus emblica* extract, and pure PVP film.

FTIR was used to investigate the structural characterization and chemical interactions in composite dressing films. The neat PVP film exhibited a broad band O-H stretching of hydrogen-bonded water at roughly 3440 cm^−1^, an asymmetric CH_2_ stretching peak of the pyrrolidone ring at 2960 cm^−1^, a symmetric CH_2_ stretching peak of the backbone chain at 2922 cm^−1^, and a typical C=O stretching peak in the pyrrolidone ring at 1656 cm^−1^, as shown in Figure 5. At 1433 and 1284 cm^−1^, C-H bending and C-N stretching, respectively, were found. The 1018 and 574 cm^−1^ peaks were classified as CH_2_ rock and N-C=O bending, respectively. Because polyphenols, flavonoids, tannins, and vitamins make up most of the compounds in *Phyllanthus emblica* fruit, the crude fruit extract showed peaks corresponding to O-H stretching, aromatic C-H stretching, C=O stretching, and aromatic C=C bending at 3342, 2976, 1722, and 1600 cm^−1^, respectively. Peaks of C-H bending from 1300–1450 cm^−1^; C-O stretching at 1207, 1081, and 1037 cm^−1^; and C-C stretching at 876 cm^−1^ were also seen in the crude extract. Most of the distinctive peak patterns in the composite dressing films were comparable to those in the pristine PVP, showing that the loaded fruit extract and AgNPs did not generate any chemical alterations to PVP. Interestingly, at 1722 and 1600 cm^−1^, the C=O stretching and aromatic C=C bending of the fruit extract manifested as shoulders close to a C=O stretching peak in the pyrrolidone ring of PVP at 1656 cm^−1^. This also resulted in a somewhat broader and higher peak at 1656 cm^−1^. Besides, the peak at 1018 cm^−1^ resulting from the CH_2_ rock of PVP vanished from the composite film spectra after loading with the extract and AgNPs, while the C-O stretching bands of the extract at 1081 and 1037 cm^−1^ appeared and became progressively higher, broader, and shifted to 1091 cm^−1^. Thus, the extract was effectively loaded into the composite dressing films and may have interacted with PVP and AgNPs via the oxygen-containing functional groups, as indicated by these results.

#### 3.1.6. Film Morphology Analysis

**Figure 6:**
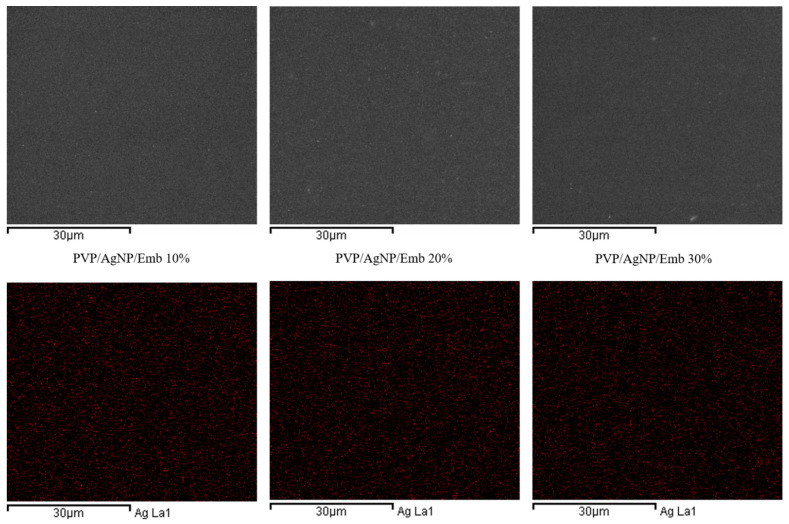
SEM images of spray-on dressing films loaded with different amounts of *Phyllanthus emblica* extract at 2500× magnification and their corresponding EDX mapping of elemental silver.

**Figure 7:**
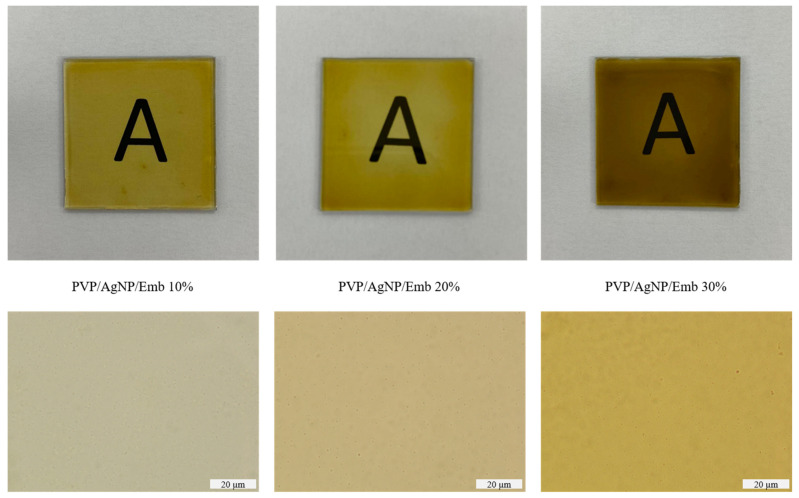
Optical images of spray-on films loaded with different amounts of *Phyllanthus emblica* extract on glass slides and their surface morphology obtained using a digital microscope at 100× magnification.

A scanning electron microscope (SEM) was used to examine the surface morphology of spray-on dressing films containing various quantities of *Phyllanthus emblica* extract. Figure 6 shows that all of the spray-on dressing films have smooth and consistent surfaces with no indications of aggregation. The EDX elemental mapping also shows the presence of metallic silver, which is color-coded in red and well-distributed throughout the images, signifying that the silver nanoparticles were uniformly dispersed and spread throughout the PVP films. The spray-on solution may be used on any shape or sized location. The application of spray-on films to the glass slides is shown in Figure 7. The spray quickly formed a clear, fast-drying layer, which was consistent without fractures. The dressing films also had smooth and uniform surfaces, as indicated by a morphological study using a digital microscope at 100× magnification.

### 3.2. In Vitro Release Study

**Figure 8:**
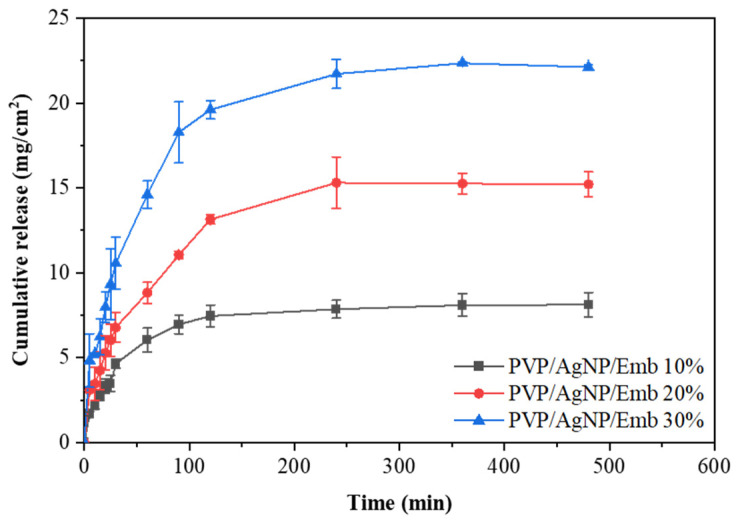
Cumulative release of *Phyllanthus emblica* extract from spray-on dressing films loaded with different amounts of extract.

In vitro Franz diffusion assays were used to investigate the in vitro release of the *Phyllanthus emblica* extract from the spray-on dressing films. Figure 8 illustrates the cumulative release of fruit extract from films loaded with three distinct quantities of *Phyllanthus emblica* extract. Increased discharge of the extract was seen as the loading quantity increased. The dressing films revealed a rapid release at an early step in all cases, owing to the rapid onset of the loosely bound or encapsulated fruit extract adjacent to the dressing film surfaces. After a period of rapid release, the extract imbedded in the polymeric gel film was progressively discharged in a regulated and sustained way until it reached a plateau at roughly 4 h. This implies that the sprayed gel film could deliver the extract to the wound bed quickly and effectively, thus boosting the healing process. 

The Ritger–Peppas model, which is often used to explain the release mechanism of polymeric matrix-based systems, was chosen to characterize the tested system’s release mechanism since it considers many mechanisms at once, such as diffusion, matrix swelling, and dissolution [48]. The coefficients of determination (R^2^) for the dressing films loaded with 10%, 20%, and 30% *Phyllanthus emblica* extract were reported to be 0.9924, 0.9939, and 0.9919, respectively, after fitting the release data with the Ritger–Peppas model, indicating that the release kinetics were very well fitted to the selected model. To determine the mechanism responsible for the release, the diffusion exponent (n) was utilized. The releasing mechanism follows a Fickian diffusion at *n* ≤ 0.5. In this instance, the drug diffuses through a non-swollen matrix, and the discharge is dependent on a drug concentration gradient between the polymeric matrix and the release medium [49]. The release mechanism is specified as case II transport when *n* = 1.00. The dissolution or swelling of the polymer layer causes the release in this case. The release mechanism is regarded as an anomalous non-Fickian transport when 0.50 < *n* < 1.00. Both diffusion and dissolution/swelling of the polymer matrix control drug release in this situation. For the dressing films loaded with 10%, 20%, and 30% of the extract, the exponent *n* values were 0.61, 0.53, and 0.54, respectively, in this study. These values range between 0.50 and 1.00, showing that the release is regulated by a mix of diffusion and matrix-swelling processes. 

### 3.3. Total Phenolic Content and In Vitro Antioxidant Test

Reactive oxygen species (ROS) are extremely reactive oxidizing agents generated primarily in mitochondria from molecular oxygen molecules. ROS have been discovered to have a role in wound healing at several stages. It has been shown that a modest amount of these active species is advantageous in protecting cells against infection. Excessive amounts, on the other hand, could cause oxidative stress by having negative effects on tissues, impeding wound healing [31]. Antioxidants are chemical substances that impede the development of radical chain reactions, thus inhibiting free radicals or reactive oxygen species. As a result, antioxidants are thought to aid wound healing by working to protect against oxidative stress and inflammation [41].

Plant-derived polyphenolic compounds have been shown to exhibit significant antioxidant properties in vitro and in vivo. These chemicals have been revealed to be major components in *Phyllanthus emblica* fruit extract [50,51,52]. As a consequence, they are thought to play a significant role in the actions of current antioxidant spray dressings. Table 1 shows the total phenolic content and antioxidant activity of a dressing spray comprising *Phyllanthus emblica* fruit extract. In sprays comprising 10%, 20%, and 30% fruit extract, the overall phenolic contents were found to be 1.56 ± 0.13, 2.73 ± 0.11, and 3.86 ± 0.17 GAE mg/g, respectively. The total phenolic content of the extract increases as the loading quantity of the extract increases, according to these data. DPPH test was used to assess the free radical scavenging abilities of the spray dressing. When the loading quantity of the *Phyllanthus emblica* fruit extract increases, the proportion of scavenging activity increases from 49.02 ± 3.35% to 64.56 ± 2.44% and 69.22 ± 0.26%, resembling the phenolic content. This indicates that the antioxidant activity of the spray dressings is attributable to the *Phyllanthus emblica* extract.

### 3.4. Antibacterial Test

**Figure 9:**
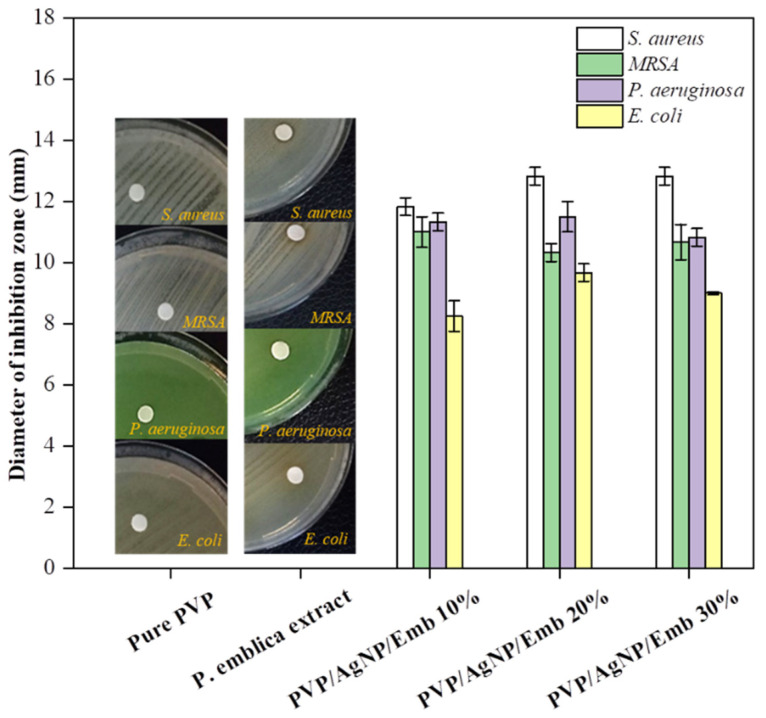
Antibacterial activity of PVP, *Phyllanthus emblica* extract, and *Phyllanthus emblica* extract-loaded spray-on dressing films against *S. aureus*, MRSA, *P. aeruginosa,* and *E. coli*.

The disk diffusion approach was used to test the antibacterial activity of *P. emblica* extract/silver nanoparticles/polyvinylpyrrolidone spray-on dressing. *P. aeruginosa, E. coli, S. aureus*, and the resistant MRSA were chosen as typical Gram-negative and Gram-positive bacterial strains often associated with wound infections. The clear inhibition zones were then investigated, with the results shown in Figure 9. PVP spray and *Phyllanthus emblica* extract had no distinct inhibition zones against any of the tested strains as a control, suggesting that PVP and the extract exhibited no antibacterial activity. This is contrary to the spray-on dressing, which showed obvious inhibitory zones against all bacterial strains in all of the formulations tested, each with various loading quantities of the fruit extract. This suggests that silver nanoparticles are the cause of bacterial growth inhibition. However, there were no substantial increments in the width of the inhibition zone between the strains, showing that the percentage of *Phyllanthus emblica* extract used had no effect on the antibacterial properties of the current spray-on dressing. 

### 3.5. In Vitro Cytotoxicity

**Figure 10:**
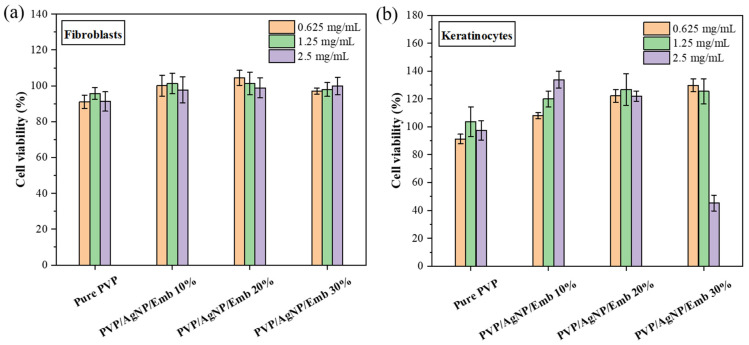
Cytotoxicity effects of PVP and dressing sprays at different loading amounts of *Phyllanthus emblica* extract on (**a**) human dermal fibroblasts and (**b**) human keratinocytes. Each formulation was tested at three concentrations (0.625, 1.25, and 2.5 mg/mL).

The MTS test was used to assess the in vitro cytotoxicity of the *Phyllanthus emblica* extract/silver nanoparticles/polyvinylpyrrolidone spray-on dressing on human dermal fibroblast (HDFa) and keratinocyte (HaCat) cells. After 24 h of incubation with the test samples at three distinct concentrations (0.63, 1.25, and 2.5 mg/mL), the viability of the tested cells was measured (Figure 10).

In this test, cell viability percentages of more than 80% were deemed non-toxic. The viability of the fibroblasts was greater than 90% at every concentration of the test samples, as illustrated in Figure 10a, demonstrating that the current spray-on dressing was non-toxic to fibroblast cells. Although the *Phyllanthus emblica* extract has high antioxidant activity, the loading quantity can have an adverse impact on the viability of the evaluated cells, specifically keratinocytes. Figure 10b demonstrates this notion. The viability of the keratinocyte declines to less than 50% when the tested dose of the spray-on dressing containing 30% *Phyllanthus emblica* extract is increased to 2.5 mg/mL. This implies that, when the *Phyllanthus emblica* extract loading is less than 20%, the spray-on treatment is biocompatible and suitable for use as a material for wound dressings. It should be noted that at this loading amount antioxidant and antibacterial activity is equivalent to that of a 30% loading level. As a result, 20% of the *Phyllanthus emblica* extract is recommended for use in spray-on dressings.

## 4. Conclusions

Utilizing PVP as an adhesive film-former, silver nanoparticles as a broad-spectrum antimicrobial agent, and *Phyllanthus emblica* extract as a natural antioxidant, an antibacterial spray-on wound dressing was successfully developed in this study. The spray-on solution was made in one pot, employing the natural fruit extract from *Phyllanthus emblica* for the green synthesis of silver nanoparticles. The adhesive hydrogel film forms quickly after being sprayed on the wound surface and serves as a protective barrier to keep fluids in the wound bed while keeping germs out. The *Phyllanthus emblica* extract, in addition to acting as a biogenic reducing agent, also acts as an antioxidant, helping to protect against wound oxidative stress and modulating inflammation and facilitating the healing of wounds. The extract was discharged from the polymer gel matrix in a controlled and sustained manner because it was confined inside the PVP film. The incorporation of silver nanoparticles in the spray provided antibacterial activity against Gram-positive and Gram-negative bacteria, such as *S. aureus, P. aeruginosa,* and *E. coli*, as well as resistant strains such as MRSA. In vitro cytotoxicity tests demonstrated that the dressing film was biocompatible with the evaluated human skin fibroblasts and human keratinocytes except for the formula with a high loading content of the *Phyllanthus emblica* extract (30%) at the maximum dose of the sample (2.5 mg/mL). The proposed spray-on dressing seems to have considerable potential for use as an antibacterial wound dressing, based on these findings.

## Figures and Tables

**Table 1:** Antioxidant activity and total phenolic content of neat PVP and dressing spray containing different amounts of *Phyllanthus emblica* extract.

Sample	Total Phenolic Content (mg GAE/g Extract)	DPPH Scavenging (%)
PVP	0	0
PVP/AgNP/Emb 10%	1.56 ± 0.13	49.02 ± 3.35
PVP/AgNP/Emb 20%	2.73 ± 0.11	64.56 ± 2.44
PVP/AgNP/Emb 30%	3.86 ± 0.17	69.22 ± 0.26

## Data Availability

Not applicable.
